# 3,4-Dimeth­oxy-4′-nitro-1,1′-biphen­yl

**DOI:** 10.1107/S1600536812013657

**Published:** 2012-04-04

**Authors:** Xin-Min Li, Yan-Jun Hou, Wen-Yi Chu, Zhi-Zhong Sun

**Affiliations:** a College of Chemistry and Materials Science, Heilongjiang University, Harbin 150080, People’s Republic of China

## Abstract

The title compound, C_14_H_13_NO_4_, was prepared through a palladium-catalysed Suzuki–Miyaura coupling reaction. The asymmetric unit comprises two mol­ecules related by pseudo-inversion symmetry. The dihedral angles between the benzene rings in the two mol­ecules are 44.30 (6) and 48.50 (6)° while those between the benzene ring and the nitro group are 6.54 (13) and 5.73 (10)°. The crystal packing is defined only by Van der Waals inter­actions.

## Related literature

For general background to the synthesis and properties of 3,4-dimeth­oxy-4′-nitro-1,1′-biphenyl, see: Suzuki (1999 ▶); Razler *et al.* (2009 ▶); Hou *et al.* (2011 ▶); Li *et al.* (2012 ▶). For the biological activity of biphenyl derivatives, see: Kimpe *et al.* (1996 ▶).
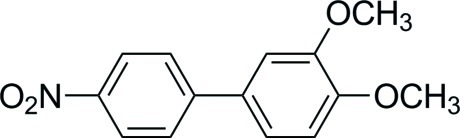


## Experimental

### 

#### Crystal data

C_14_H_13_NO_4_
*M* *_r_* = 259.25
Monoclinic, 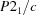

*a* = 16.2714 (14) Å
*b* = 7.6529 (7) Å
*c* = 20.2448 (18) Å
β = 91.691 (1)°
*V* = 2519.9 (4) Å^3^
*Z* = 8
Mo *K*α radiation
μ = 0.10 mm^−1^
*T* = 295 K
0.28 × 0.24 × 0.22 mm

#### Data collection

Bruker APEXII CCD detector diffractometer
Absorption correction: multi-scan (*SADABS*; Sheldrick, 1996) ▶ *T*_min_ = 0.972, *T*_max_ = 0.978
15429 measured reflections
4401 independent reflections
3201 reflections with *I* > 2σ(*I*)
*R*_int_ = 0.025

#### Refinement

*R*[*F* ^2^ > 2σ(*F* ^2^)] = 0.038
*wR*(*F* ^2^) = 0.104
*S* = 1.05
4401 reflections
348 parameters
1 restraint
H-atom parameters constrained
Δρ_max_ = 0.13 e Å^−3^
Δρ_min_ = −0.13 e Å^−3^

### 

Data collection: *APEX2* (Bruker, 2004 ▶); cell refinement: *SAINT* (Bruker, 2004 ▶); data reduction: *SAINT*; program(s) used to solve structure: *SHELXS97* (Sheldrick, 2008 ▶); program(s) used to refine structure: *SHELXL97* (Sheldrick, 2008 ▶); molecular graphics: *SHELXTL* (Sheldrick, 2008 ▶); software used to prepare material for publication: *publCIF* (Westrip, 2010 ▶).

## Supplementary Material

Crystal structure: contains datablock(s) I, global. DOI: 10.1107/S1600536812013657/kp2400sup1.cif

Supplementary material file. DOI: 10.1107/S1600536812013657/kp2400Isup2.cdx

Structure factors: contains datablock(s) I. DOI: 10.1107/S1600536812013657/kp2400Isup3.hkl

Supplementary material file. DOI: 10.1107/S1600536812013657/kp2400Isup4.cml

Additional supplementary materials:  crystallographic information; 3D view; checkCIF report

## Figures and Tables

**Fig. 1.**
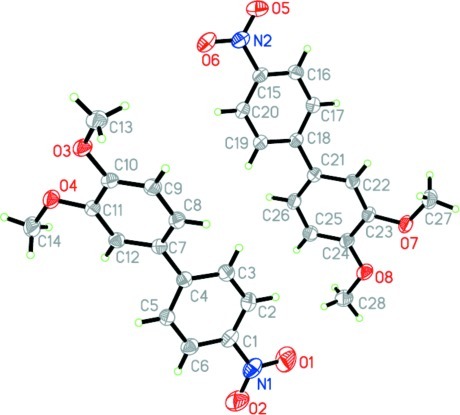
The two molecules of asymmetric unit (I) with displacement ellipsoids drawn at the 50% probability level.

**Fig. 2.**
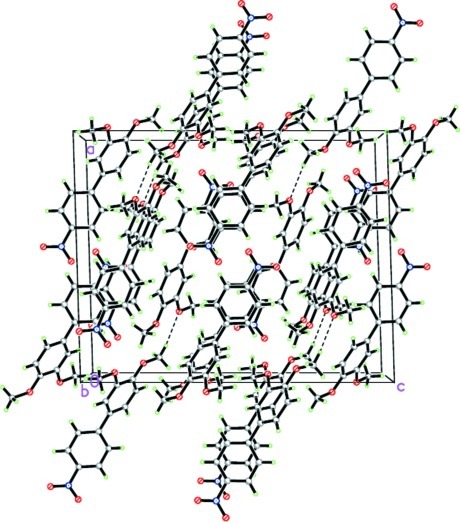
Van der Waals interactions dominat the crystal packing.

## supplementary crystallographic information

### Comment

The palladium-catalyzed Suzuki-Miyaura coupling reaction attracts a considerable interest. The biological activity of biphenyl derivatives (Suzuki, 1999; Razler *et al.*, 2009; Kimpe *et al.*, 1996; Hou *et al.*, 2011; Li *et al.*, 2012) has been described in the literature. We have prepared 3,4-dimethoxy-4'-nitro-1,1'-biphenyl as a potentially active antiviral compound. In the title compound there are two molecules in an asymmetric unit ( Fig. 1). The dihedral angle between the benzene rings (C1-C2-C3-C4-C5-C6) and (C7-C8-C9-C10-C11-C12) is 44.30 (6)°; (C15-C16-C17-C18-C19-C20) and (C21-C22-C23-C24-C25-C26) is 48.50 (6)°. The dihedral angle between the benzene ring (C1-C2-C3-C4-C5-C6) and nitro group (N1-O1-O2) is 6.54 (13)°. The dihedral angle between the benzene ring (C15-C16-C17-C18 C19-C20) and nitro group (N2-O5-O6) is 5.73 (10)°. Van der Waals interactions dominate the crystal packing (Fig. 2).

### Experimental

To a solution of 1-bromo-4-nitrobenzene (5 mmol) and 3,4-dimethoxyphenylboronic acid (6 mmol) in 20 mL water and 20 mL methanol, Pd(OAc)_2_ (5 mmol) and K_2_CO_3_ (10 mmol) were added. After stirring the reaction mixture for 6 h at 323 K, the aqueous phases were extracted with 100 mL ethyl acetate. The organic extracts were washed with 200 mL saturated aqueous sodium chlorid, dried over sodium sulfate, filtered, and concentrated under reduced pressure. The resulting crude material was purified *via* silica gel chromatography (petroleum ether) to afford a translucent solid in a yield of 63%. Crystals suitable for single-crystal X-ray diffraction were obtained by recrystallisation from methanol at room temperature in a total yield of 44%. Analysis found: C 64.9, H 5.2, N 5.5%; C_14_H_13_NO_4_ requires: 64.9, H 5.1, N 5.4%. ^1^H NMR (400 MHz, CDCl_3_) 8.34 - 8.23 (m, 2H), 7.77 - 7.66 (m, 2H), 7.21 (dd, *J* = 8.3, 2.2 Hz, 1H), 7.13 (d, *J* = 2.2 Hz, 1H), 6.99 (d, *J* = 8.3 Hz, 1H), 3.97 (s, 3H), 3.95 (s, 3H).

### Refinement

H atoms bound to C atoms were placed in calculated positions and treated as riding on their parent atoms. C—H distances are in the range 0.93–0.96 Å. *U*_iso_(H) values were constrained to be 1.2*U*_eq_(C) (aromatic H atoms) [1.5*U*_eq_(C) for methyl H atoms].

### Crystal data

**Table:**

C_14_H_13_NO_4_	*F*(000) = 1088
*M**_r_* = 259.25	*D*_x_ = 1.367 Mg m^−^^3^
Monoclinic, *P*2_1_/*c*	Mo *K*α radiation, λ = 0.71073 Å
Hall symbol: -P 2ybc	Cell parameters from 3878 reflections
*a* = 16.2714 (14) Å	θ = 2.3–24.0°
*b* = 7.6529 (7) Å	µ = 0.10 mm^−^^1^
*c* = 20.2448 (18) Å	*T* = 295 K
β = 91.691 (1)°	Block, colourless
*V* = 2519.9 (4) Å^3^	0.28 × 0.24 × 0.22 mm
*Z* = 8	

### Data collection

**Table:**

Bruker APEXII CCD detector diffractometer	4401 independent reflections
Radiation source: fine-focus sealed tube	3201 reflections with *I* > 2σ(*I*)
Graphite monochromator	*R*_int_ = 0.025
phi and ω scans	θ_max_ = 25.1°, θ_min_ = 2.3°
Absorption correction: multi-scan (*SADABS*; Sheldrick, 1996)	*h* = −19→19
*T*_min_ = 0.972, *T*_max_ = 0.978	*k* = −9→8
15429 measured reflections	*l* = −24→24

### Refinement

**Table:**

Refinement on *F*^2^	Secondary atom site location: difference Fourier map
Least-squares matrix: full	Hydrogen site location: inferred from neighbouring sites
*R*[*F*^2^ > 2σ(*F*^2^)] = 0.038	H-atom parameters constrained
*wR*(*F*^2^) = 0.104	*w* = 1/[σ^2^(*F*_o_^2^) + (0.050*P*)^2^ + 0.2279*P*] where *P* = (*F*_o_^2^ + 2*F*_c_^2^)/3
*S* = 1.05	(Δ/σ)_max_ = 0.001
4401 reflections	Δρ_max_ = 0.13 e Å^−^^3^
348 parameters	Δρ_min_ = −0.13 e Å^−^^3^
1 restraint	Extinction correction: *SHELXL97* (Sheldrick, 2008), Fc^*^=kFc[1+0.001xFc^2^λ^3^/sin(2θ)]^-1/4^
Primary atom site location: structure-invariant direct methods	Extinction coefficient: 0.0058 (7)

### Special details

**Table:**

Geometry. All e.s.d.'s (except the e.s.d. in the dihedral angle between two l.s. planes) are estimated using the full covariance matrix. The cell e.s.d.'s are taken into account individually in the estimation of e.s.d.'s in distances, angles and torsion angles; correlations between e.s.d.'s in cell parameters are only used when they are defined by crystal symmetry. An approximate (isotropic) treatment of cell e.s.d.'s is used for estimating e.s.d.'s involving l.s. planes.
Refinement. Refinement of *F*^2^ against ALL reflections. The weighted *R*-factor *wR* and goodness of fit *S* are based on *F*^2^, conventional *R*-factors *R* are based on *F*, with *F* set to zero for negative *F*^2^. The threshold expression of *F*^2^ > σ(*F*^2^) is used only for calculating *R*-factors(gt) *etc*. and is not relevant to the choice of reflections for refinement. *R*-factors based on *F*^2^ are statistically about twice as large as those based on *F*, and *R*- factors based on ALL data will be even larger.

### Fractional atomic coordinates and isotropic or equivalent isotropic displacement parameters (Å^2^)

**Table:**

	*x*	*y*	*z*	*U*_iso_*/*U*_eq_	
O3	0.22448 (7)	0.63104 (14)	0.23783 (5)	0.0571 (3)	
O4	0.27112 (7)	0.37343 (14)	0.31153 (5)	0.0574 (3)	
C7	0.46025 (9)	0.64220 (19)	0.32340 (7)	0.0426 (4)	
N2	0.54229 (9)	0.36706 (18)	−0.07790 (8)	0.0591 (4)	
C15	0.62073 (9)	0.37383 (19)	−0.04093 (8)	0.0472 (4)	
O8	1.05775 (7)	0.44259 (16)	0.18817 (6)	0.0669 (4)	
C12	0.40531 (9)	0.50427 (19)	0.33426 (7)	0.0435 (4)	
H12	0.4216	0.4118	0.3614	0.052*	
C11	0.32767 (9)	0.50402 (19)	0.30529 (7)	0.0427 (4)	
C19	0.69556 (9)	0.3404 (2)	0.06001 (7)	0.0500 (4)	
H19	0.6968	0.3150	0.1050	0.060*	
C21	0.84677 (9)	0.3872 (2)	0.06898 (7)	0.0463 (4)	
C18	0.76892 (9)	0.37979 (19)	0.02932 (7)	0.0448 (4)	
C5	0.56002 (10)	0.5714 (2)	0.41620 (7)	0.0494 (4)	
H5	0.5166	0.5268	0.4399	0.059*	
C22	0.90164 (9)	0.5261 (2)	0.06122 (7)	0.0483 (4)	
H22	0.8909	0.6094	0.0287	0.058*	
C8	0.43361 (10)	0.7800 (2)	0.28437 (7)	0.0511 (4)	
H8	0.4688	0.8733	0.2771	0.061*	
O7	1.02683 (7)	0.67505 (15)	0.09849 (6)	0.0657 (3)	
C4	0.54441 (9)	0.63894 (18)	0.35348 (7)	0.0435 (4)	
N1	0.78555 (10)	0.6281 (2)	0.43826 (9)	0.0682 (4)	
C10	0.30184 (9)	0.6445 (2)	0.26520 (7)	0.0455 (4)	
C26	0.86516 (10)	0.2620 (2)	0.11649 (8)	0.0551 (4)	
H26	0.8296	0.1683	0.1219	0.066*	
O6	0.48073 (7)	0.31992 (18)	−0.04893 (7)	0.0762 (4)	
C23	0.97134 (9)	0.5411 (2)	0.10119 (7)	0.0488 (4)	
C1	0.70214 (10)	0.6327 (2)	0.40854 (8)	0.0518 (4)	
C6	0.63842 (10)	0.5688 (2)	0.44434 (8)	0.0551 (4)	
H6A	0.6478	0.5246	0.4867	0.066*	
C2	0.68964 (10)	0.7008 (2)	0.34608 (8)	0.0551 (4)	
H2	0.7335	0.7436	0.3225	0.066*	
C16	0.69146 (10)	0.4127 (2)	−0.07351 (8)	0.0532 (4)	
H16	0.6896	0.4359	−0.1186	0.064*	
C24	0.98862 (9)	0.4140 (2)	0.14981 (8)	0.0519 (4)	
C20	0.62188 (10)	0.3381 (2)	0.02565 (8)	0.0513 (4)	
H20	0.5734	0.3128	0.0469	0.062*	
C9	0.35498 (10)	0.7815 (2)	0.25574 (7)	0.0522 (4)	
H9	0.3381	0.8760	0.2299	0.063*	
C3	0.61105 (10)	0.7044 (2)	0.31925 (8)	0.0515 (4)	
H3	0.6020	0.7516	0.2773	0.062*	
C17	0.76529 (10)	0.4165 (2)	−0.03812 (7)	0.0525 (4)	
H17	0.8133	0.4440	−0.0596	0.063*	
O5	0.54103 (9)	0.4053 (2)	−0.13626 (7)	0.0922 (5)	
O2	0.79437 (9)	0.5829 (2)	0.49556 (8)	0.0999 (5)	
C25	0.93586 (10)	0.2746 (2)	0.15612 (8)	0.0579 (4)	
H25	0.9478	0.1881	0.1872	0.069*	
C13	0.19155 (11)	0.7810 (2)	0.20433 (9)	0.0650 (5)	
H13A	0.1897	0.8775	0.2346	0.097*	
H13B	0.1370	0.7556	0.1877	0.097*	
H13C	0.2259	0.8108	0.1682	0.097*	
C14	0.29085 (11)	0.2330 (2)	0.35539 (9)	0.0687 (5)	
H14A	0.3397	0.1754	0.3412	0.103*	
H14B	0.2461	0.1511	0.3552	0.103*	
H14C	0.3000	0.2778	0.3993	0.103*	
O1	0.84249 (9)	0.6716 (2)	0.40461 (9)	0.1068 (6)	
C27	1.00852 (12)	0.8142 (2)	0.05341 (10)	0.0764 (6)	
H27A	1.0046	0.7688	0.0092	0.115*	
H27B	1.0515	0.9000	0.0563	0.115*	
H27C	0.9572	0.8671	0.0644	0.115*	
C28	1.07699 (12)	0.3182 (3)	0.23870 (9)	0.0782 (6)	
H28A	1.0325	0.3122	0.2688	0.117*	
H28B	1.1264	0.3532	0.2623	0.117*	
H28C	1.0850	0.2055	0.2191	0.117*	

### Atomic displacement parameters (Å^2^)

**Table:**

	*U*^11^	*U*^22^	*U*^33^	*U*^12^	*U*^13^	*U*^23^
O3	0.0494 (7)	0.0586 (7)	0.0624 (7)	−0.0024 (5)	−0.0154 (5)	0.0107 (5)
O4	0.0507 (7)	0.0524 (7)	0.0685 (7)	−0.0100 (5)	−0.0097 (5)	0.0158 (5)
C7	0.0435 (9)	0.0446 (9)	0.0396 (8)	0.0001 (7)	−0.0006 (6)	−0.0010 (6)
N2	0.0560 (9)	0.0477 (9)	0.0725 (10)	0.0013 (7)	−0.0162 (7)	−0.0033 (7)
C15	0.0448 (9)	0.0403 (9)	0.0560 (9)	−0.0006 (7)	−0.0089 (7)	−0.0033 (7)
O8	0.0516 (7)	0.0705 (8)	0.0772 (8)	−0.0084 (6)	−0.0228 (6)	0.0150 (6)
C12	0.0463 (9)	0.0415 (9)	0.0426 (8)	0.0017 (7)	−0.0024 (7)	0.0031 (6)
C11	0.0432 (9)	0.0425 (9)	0.0423 (8)	−0.0033 (7)	−0.0002 (6)	−0.0006 (6)
C19	0.0486 (10)	0.0551 (10)	0.0461 (8)	−0.0081 (8)	−0.0011 (7)	−0.0007 (7)
C21	0.0414 (9)	0.0483 (10)	0.0493 (9)	−0.0022 (7)	−0.0006 (7)	−0.0012 (7)
C18	0.0436 (9)	0.0405 (9)	0.0502 (9)	−0.0035 (7)	−0.0016 (7)	−0.0034 (7)
C5	0.0462 (9)	0.0523 (10)	0.0496 (9)	−0.0051 (7)	−0.0016 (7)	0.0054 (7)
C22	0.0430 (9)	0.0499 (10)	0.0517 (9)	−0.0015 (7)	−0.0019 (7)	0.0047 (7)
C8	0.0527 (10)	0.0487 (10)	0.0516 (9)	−0.0075 (8)	−0.0047 (7)	0.0076 (7)
O7	0.0504 (7)	0.0627 (8)	0.0830 (8)	−0.0178 (6)	−0.0166 (6)	0.0186 (6)
C4	0.0456 (9)	0.0389 (9)	0.0460 (8)	0.0002 (7)	0.0003 (7)	−0.0022 (6)
N1	0.0514 (10)	0.0587 (10)	0.0934 (12)	0.0013 (8)	−0.0146 (9)	−0.0039 (8)
C10	0.0435 (9)	0.0501 (10)	0.0424 (8)	0.0009 (7)	−0.0054 (7)	−0.0001 (7)
C26	0.0484 (10)	0.0533 (11)	0.0632 (10)	−0.0087 (8)	−0.0033 (8)	0.0056 (8)
O6	0.0476 (7)	0.0842 (10)	0.0961 (10)	−0.0051 (6)	−0.0101 (6)	−0.0093 (7)
C23	0.0391 (9)	0.0491 (10)	0.0582 (9)	−0.0053 (7)	−0.0010 (7)	0.0010 (7)
C1	0.0425 (9)	0.0426 (9)	0.0698 (11)	0.0013 (7)	−0.0076 (8)	−0.0072 (8)
C6	0.0570 (11)	0.0499 (10)	0.0575 (10)	−0.0012 (8)	−0.0116 (8)	0.0039 (8)
C2	0.0462 (10)	0.0527 (10)	0.0667 (11)	−0.0029 (8)	0.0064 (8)	−0.0004 (8)
C16	0.0598 (11)	0.0509 (10)	0.0486 (9)	−0.0040 (8)	−0.0051 (8)	0.0041 (7)
C24	0.0410 (9)	0.0563 (10)	0.0579 (10)	−0.0006 (8)	−0.0062 (7)	0.0037 (8)
C20	0.0432 (9)	0.0510 (10)	0.0598 (10)	−0.0082 (7)	0.0012 (7)	−0.0054 (8)
C9	0.0558 (10)	0.0487 (10)	0.0514 (9)	−0.0024 (8)	−0.0086 (8)	0.0114 (7)
C3	0.0512 (10)	0.0537 (10)	0.0495 (9)	−0.0013 (8)	0.0023 (7)	0.0029 (7)
C17	0.0487 (10)	0.0553 (10)	0.0535 (9)	−0.0068 (8)	0.0019 (7)	0.0019 (7)
O5	0.0848 (10)	0.1121 (12)	0.0778 (9)	−0.0064 (9)	−0.0319 (8)	0.0203 (9)
O2	0.0747 (10)	0.1221 (13)	0.1007 (12)	−0.0039 (9)	−0.0361 (9)	0.0143 (10)
C25	0.0542 (11)	0.0555 (11)	0.0633 (10)	−0.0028 (8)	−0.0086 (8)	0.0128 (8)
C13	0.0570 (11)	0.0665 (12)	0.0703 (11)	0.0061 (9)	−0.0153 (9)	0.0138 (9)
C14	0.0677 (12)	0.0608 (12)	0.0773 (12)	−0.0131 (9)	−0.0041 (10)	0.0217 (9)
O1	0.0442 (8)	0.1423 (15)	0.1336 (14)	−0.0050 (9)	−0.0012 (9)	0.0187 (11)
C27	0.0659 (13)	0.0623 (13)	0.1000 (15)	−0.0166 (10)	−0.0141 (11)	0.0240 (11)
C28	0.0622 (12)	0.0906 (15)	0.0804 (13)	−0.0033 (11)	−0.0239 (10)	0.0238 (11)

### Geometric parameters (Å, º)

**Table:**

O3—C10	1.3640 (17)	C4—C3	1.397 (2)
O3—C13	1.4295 (19)	N1—O1	1.213 (2)
O4—C11	1.3668 (17)	N1—O2	1.215 (2)
O4—C14	1.4248 (19)	N1—C1	1.469 (2)
C7—C8	1.380 (2)	C10—C9	1.376 (2)
C7—C12	1.405 (2)	C26—C25	1.386 (2)
C7—C4	1.482 (2)	C26—H26	0.9300
N2—O5	1.2167 (18)	C23—C24	1.406 (2)
N2—O6	1.2297 (18)	C1—C6	1.373 (2)
N2—C15	1.461 (2)	C1—C2	1.377 (2)
C15—C20	1.375 (2)	C6—H6A	0.9300
C15—C16	1.376 (2)	C2—C3	1.375 (2)
O8—C24	1.3655 (18)	C2—H2	0.9300
O8—C28	1.425 (2)	C16—C17	1.381 (2)
C12—C11	1.3770 (19)	C16—H16	0.9300
C12—H12	0.9300	C24—C25	1.378 (2)
C11—C10	1.404 (2)	C20—H20	0.9300
C19—C20	1.368 (2)	C9—H9	0.9300
C19—C18	1.395 (2)	C3—H3	0.9300
C19—H19	0.9300	C17—H17	0.9300
C21—C26	1.384 (2)	C25—H25	0.9300
C21—C22	1.400 (2)	C13—H13A	0.9600
C21—C18	1.481 (2)	C13—H13B	0.9600
C18—C17	1.394 (2)	C13—H13C	0.9600
C5—C6	1.382 (2)	C14—H14A	0.9600
C5—C4	1.387 (2)	C14—H14B	0.9600
C5—H5	0.9300	C14—H14C	0.9600
C22—C23	1.378 (2)	C27—H27A	0.9600
C22—H22	0.9300	C27—H27B	0.9600
C8—C9	1.389 (2)	C27—H27C	0.9600
C8—H8	0.9300	C28—H28A	0.9600
O7—C23	1.3681 (18)	C28—H28B	0.9600
O7—C27	1.428 (2)	C28—H28C	0.9600
			
C10—O3—C13	117.44 (12)	C6—C1—N1	118.57 (16)
C11—O4—C14	118.00 (12)	C2—C1—N1	119.67 (16)
C8—C7—C12	118.24 (14)	C1—C6—C5	118.66 (15)
C8—C7—C4	121.26 (14)	C1—C6—H6A	120.7
C12—C7—C4	120.50 (13)	C5—C6—H6A	120.7
O5—N2—O6	122.89 (15)	C3—C2—C1	118.73 (15)
O5—N2—C15	118.57 (15)	C3—C2—H2	120.6
O6—N2—C15	118.52 (15)	C1—C2—H2	120.6
C20—C15—C16	121.71 (14)	C15—C16—C17	118.90 (14)
C20—C15—N2	118.78 (14)	C15—C16—H16	120.6
C16—C15—N2	119.50 (14)	C17—C16—H16	120.6
C24—O8—C28	117.46 (13)	O8—C24—C25	125.20 (14)
C11—C12—C7	120.96 (13)	O8—C24—C23	115.57 (14)
C11—C12—H12	119.5	C25—C24—C23	119.23 (14)
C7—C12—H12	119.5	C19—C20—C15	118.83 (15)
O4—C11—C12	124.98 (13)	C19—C20—H20	120.6
O4—C11—C10	114.99 (13)	C15—C20—H20	120.6
C12—C11—C10	120.03 (13)	C10—C9—C8	120.71 (14)
C20—C19—C18	121.65 (14)	C10—C9—H9	119.6
C20—C19—H19	119.2	C8—C9—H9	119.6
C18—C19—H19	119.2	C2—C3—C4	121.56 (15)
C26—C21—C22	118.55 (14)	C2—C3—H3	119.2
C26—C21—C18	120.96 (14)	C4—C3—H3	119.2
C22—C21—C18	120.43 (14)	C16—C17—C18	121.00 (15)
C17—C18—C19	117.91 (14)	C16—C17—H17	119.5
C17—C18—C21	122.30 (14)	C18—C17—H17	119.5
C19—C18—C21	119.76 (13)	C24—C25—C26	120.57 (15)
C6—C5—C4	121.64 (15)	C24—C25—H25	119.7
C6—C5—H5	119.2	C26—C25—H25	119.7
C4—C5—H5	119.2	O3—C13—H13A	109.5
C23—C22—C21	120.93 (14)	O3—C13—H13B	109.5
C23—C22—H22	119.5	H13A—C13—H13B	109.5
C21—C22—H22	119.5	O3—C13—H13C	109.5
C7—C8—C9	121.01 (15)	H13A—C13—H13C	109.5
C7—C8—H8	119.5	H13B—C13—H13C	109.5
C9—C8—H8	119.5	O4—C14—H14A	109.5
C23—O7—C27	117.30 (12)	O4—C14—H14B	109.5
C5—C4—C3	117.64 (14)	H14A—C14—H14B	109.5
C5—C4—C7	121.61 (13)	O4—C14—H14C	109.5
C3—C4—C7	120.75 (13)	H14A—C14—H14C	109.5
O1—N1—O2	122.95 (17)	H14B—C14—H14C	109.5
O1—N1—C1	118.37 (17)	O7—C27—H27A	109.5
O2—N1—C1	118.67 (17)	O7—C27—H27B	109.5
O3—C10—C9	125.24 (14)	H27A—C27—H27B	109.5
O3—C10—C11	115.73 (13)	O7—C27—H27C	109.5
C9—C10—C11	119.03 (14)	H27A—C27—H27C	109.5
C21—C26—C25	120.87 (15)	H27B—C27—H27C	109.5
C21—C26—H26	119.6	O8—C28—H28A	109.5
C25—C26—H26	119.6	O8—C28—H28B	109.5
O7—C23—C22	124.86 (14)	H28A—C28—H28B	109.5
O7—C23—C24	115.32 (13)	O8—C28—H28C	109.5
C22—C23—C24	119.81 (14)	H28A—C28—H28C	109.5
C6—C1—C2	121.76 (15)	H28B—C28—H28C	109.5
			
O5—N2—C15—C20	177.20 (15)	C27—O7—C23—C24	−175.24 (15)
O6—N2—C15—C20	−4.1 (2)	C21—C22—C23—O7	−177.85 (15)
O5—N2—C15—C16	−3.9 (2)	C21—C22—C23—C24	1.0 (2)
O6—N2—C15—C16	174.84 (15)	O1—N1—C1—C6	174.19 (17)
C8—C7—C12—C11	1.8 (2)	O2—N1—C1—C6	−6.7 (2)
C4—C7—C12—C11	−178.45 (13)	O1—N1—C1—C2	−5.9 (2)
C14—O4—C11—C12	5.3 (2)	O2—N1—C1—C2	173.20 (17)
C14—O4—C11—C10	−175.43 (14)	C2—C1—C6—C5	0.8 (2)
C7—C12—C11—O4	178.05 (13)	N1—C1—C6—C5	−179.33 (14)
C7—C12—C11—C10	−1.2 (2)	C4—C5—C6—C1	−0.9 (2)
C20—C19—C18—C17	−0.3 (2)	C6—C1—C2—C3	0.0 (2)
C20—C19—C18—C21	177.71 (14)	N1—C1—C2—C3	−179.82 (14)
C26—C21—C18—C17	−139.08 (16)	C20—C15—C16—C17	−0.3 (2)
C22—C21—C18—C17	43.9 (2)	N2—C15—C16—C17	−179.21 (14)
C26—C21—C18—C19	43.0 (2)	C28—O8—C24—C25	0.1 (3)
C22—C21—C18—C19	−133.97 (16)	C28—O8—C24—C23	179.15 (15)
C26—C21—C22—C23	−1.8 (2)	O7—C23—C24—O8	0.8 (2)
C18—C21—C22—C23	175.24 (14)	C22—C23—C24—O8	−178.24 (14)
C12—C7—C8—C9	−0.9 (2)	O7—C23—C24—C25	179.87 (15)
C4—C7—C8—C9	179.31 (14)	C22—C23—C24—C25	0.9 (2)
C6—C5—C4—C3	0.2 (2)	C18—C19—C20—C15	0.7 (2)
C6—C5—C4—C7	−179.81 (14)	C16—C15—C20—C19	−0.4 (2)
C8—C7—C4—C5	143.17 (16)	N2—C15—C20—C19	178.48 (14)
C12—C7—C4—C5	−36.6 (2)	O3—C10—C9—C8	−178.02 (14)
C8—C7—C4—C3	−36.8 (2)	C11—C10—C9—C8	1.1 (2)
C12—C7—C4—C3	143.41 (15)	C7—C8—C9—C10	−0.5 (2)
C13—O3—C10—C9	−9.3 (2)	C1—C2—C3—C4	−0.8 (2)
C13—O3—C10—C11	171.54 (13)	C5—C4—C3—C2	0.7 (2)
O4—C11—C10—O3	−0.35 (19)	C7—C4—C3—C2	−179.31 (14)
C12—C11—C10—O3	178.96 (13)	C15—C16—C17—C18	0.8 (2)
O4—C11—C10—C9	−179.59 (13)	C19—C18—C17—C16	−0.5 (2)
C12—C11—C10—C9	−0.3 (2)	C21—C18—C17—C16	−178.42 (15)
C22—C21—C26—C25	0.7 (2)	O8—C24—C25—C26	177.02 (16)
C18—C21—C26—C25	−176.34 (15)	C23—C24—C25—C26	−2.0 (3)
C27—O7—C23—C22	3.7 (2)	C21—C26—C25—C24	1.2 (3)
